# Moyamoya Disease in a Six Month Caucasian Female

**DOI:** 10.7759/cureus.11983

**Published:** 2020-12-08

**Authors:** Faith D Moore, Tamer Rizk

**Affiliations:** 1 Medical Education, Dalhousie Medicine New Brunswick, Saint John, CAN; 2 Pediatric Neurology, Saint John Regional Hospital, Saint John, CAN

**Keywords:** moyamoya, pediatric seizure, stroke, seizure, cerebral arteriopathy, moyamoya disease, transient ischemic attack, pediatric neurology

## Abstract

Moyamoya disease (MMD) is a progressive cerebral arteriopathy characterized by stenosis and/or occlusion of the internal carotid arteries and the arteries around the Circle of Willis, with the development of “moyamoya” vessels, which are an attempt at revascularization at the base of the brain. In this paper we describe a 6 month, 3-week-old girl who presented with seizures and strokes due to moyamoya disease. The diagnosis of early onset MMD was made due to the magnetic resonance angiography results showing severe stenosis of the terminal/supraclinoid carotid arteries bilaterally with moyamoya vessels, and a completely novel de novo mutation in the RNF213 gene. She underwent bilateral encephaloduroarteriosynangiosis (EDAS) five months after her initial presentation and she did pretty well subsequently. She has shown no episodes suggestive of further strokes up to one year after surgery.

## Introduction

Moyamoya disease (MMD) is a progressive cerebral arteriopathy characterized by stenosis and/or occlusion of the internal carotid arteries and the arteries around the Circle of Willis, with the development of “moyamoya” vessels, which are an attempt at revascularization at the base of the brain [1]. MMD represents 22% of identified cerebral arteriopathies in pediatric stroke [2]. 

MMD in the pediatric population usually presents between the ages of 3-10, although it has been reported from the age of 2 months to 18 years, with a gender ratio of 1:1 [3]. It usually presents with repeated cerebral transient ischemic attacks with sudden hemiplegia that may alternate sides [4,5]. It is possible to diagnose with suspected MMD if there is narrowing and/or occlusion of the distal part of both internal carotids or the large arteries of the circle of willis (anterior, middle and posterior cerebral arteries) without excessive collateral vessel networks or unilateral narrowing and/or occlusion of the distal part of an internal carotid artery with an excessive collateral vessel network of vessels distal to the occluded artery [6]. To confirm a diagnosis of MMD, there must be a magnetic resonance angiography or conventional angiography showing narrowing or occlusion of the terminal portion of both internal carotid arteries, and a collateral network of small vessels distal to the occluded arteries unilaterally or bilaterally [6,7].

The etiology of MMD is not fully known, and is classified as primary moyamoya syndrome (also called MMD) if it is idiopathic, and classified as secondary moyamoya syndrome if there is an identifiable cause like Down Syndrome, neurofibromatosis, sickle cell disease, previous cranial irradiation or another cause of vasculopathy [6,8].

EEG of an awake patient with MMD can show re-build-up phenomenon, which is a return of high voltage slow waves, after 20-60 seconds of hyperventilation. This has not been found in any other condition and may act as a screening test for MMD [5]

Computed tomography scan (CT) may show an area of low density in the white matter of the temporal lobe, though the scope of the low density does not correlate to the stage of MMD [5].

MMD is rarely reported in early infancy, and even rarer in those of Caucasian descent. In this paper we describe a 6 month, 3-week-old girl who presented with seizures and strokes due to MMD.

## Case presentation

A 6-month, 3-week-old Caucasian female presented with status epilepticus. On presentation, she had 4 hours of left hemiconvulsion, but with no signs of encephalopathy. She was treated initially with benzodiazepines, phenobarbital loading and then started levetiracetam. There were no other seizures once she was admitted. 

Upon examination, she was in no distress, her gaze deviated to the left, with left-beating horizontal nystagmus, her tone and strength were normal throughout, there was no clonus. She had normal head circumference of 44 cm. She was right handed which is abnormal and may be a localizing sign for a patient of her age. 

There was no family history of seizures, developmental problems, congenital anomalies or sudden infant death.

Her white blood cells were elevated at 15.9x10^9^/L (normal range 5.1-14.9 X 10^9^/L). ALT was elevated at 617 units/L (normal range <31 U/L) and lactate was elevate at 3.4 mmol/L (normal range 0.5-2.2 mmol/L). Hemoglobin, platelets, hematocrit, magnesium, glucose, calcium, C-reactive protein, lipase, bilirubin, alkaline phosphatase, urea, creatinine, ammonia, sodium, potassium and chloride were all normal. Blood and urine cultures were negative. A red blood cell pyruvate kinase was 10.6 U/g Hb (normal range for ≥ 12 months old 6.7 - 14.3 U/g Hb, no established normal range for those <12 months). 

**Figure 1:**
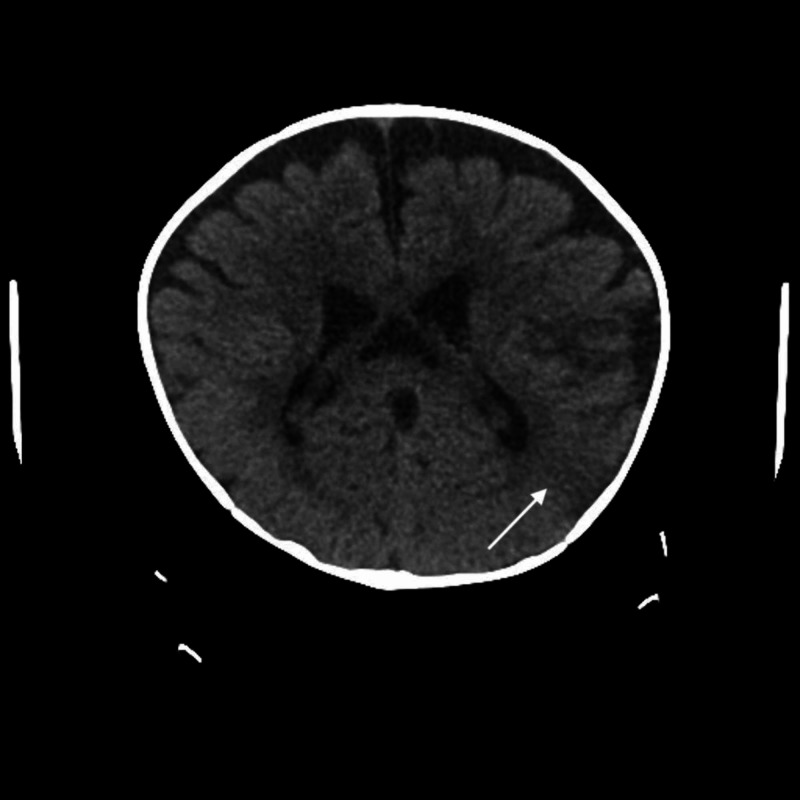
CT scan a 6-month, 3-week-old female demonstrating a left-posterior parietal lobe infarction.

MRI was attempted but unsuccessful due to motion artifact. Hence, a CT was performed and showed a left-posterior parietal lobe infarction probably from a perinatal event (Figure 1). The lab results did not indicate any cause for the stroke. A peripheral blood chromosome karyotype, the cytogenetic analysis, did not show any anomalies at the 450-550 level of resolution.

**Figure 2:**
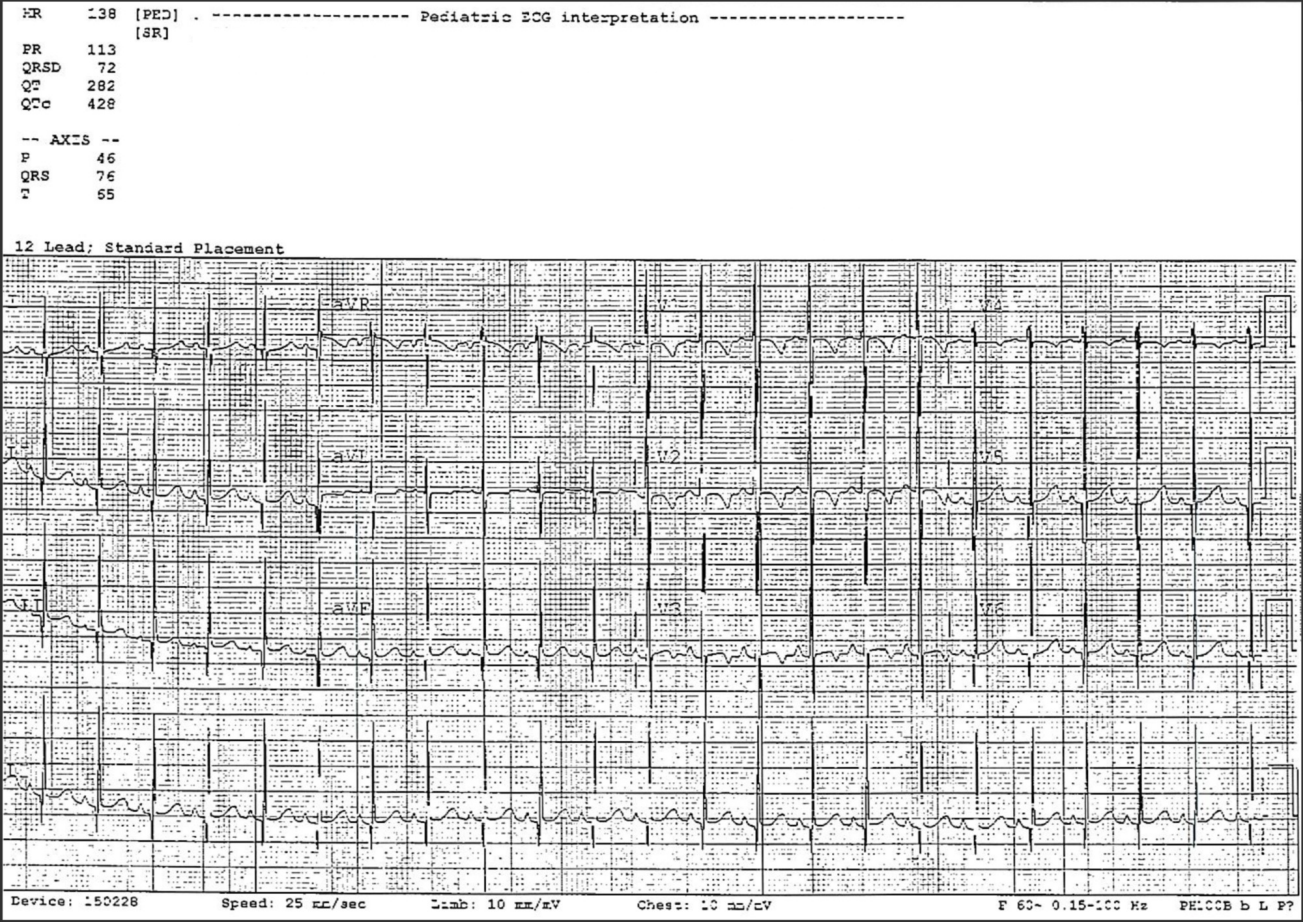
ECG of a 6-month, 3-week-old female demonstrating left ventricular hypertrophy.

**Figure 3:**
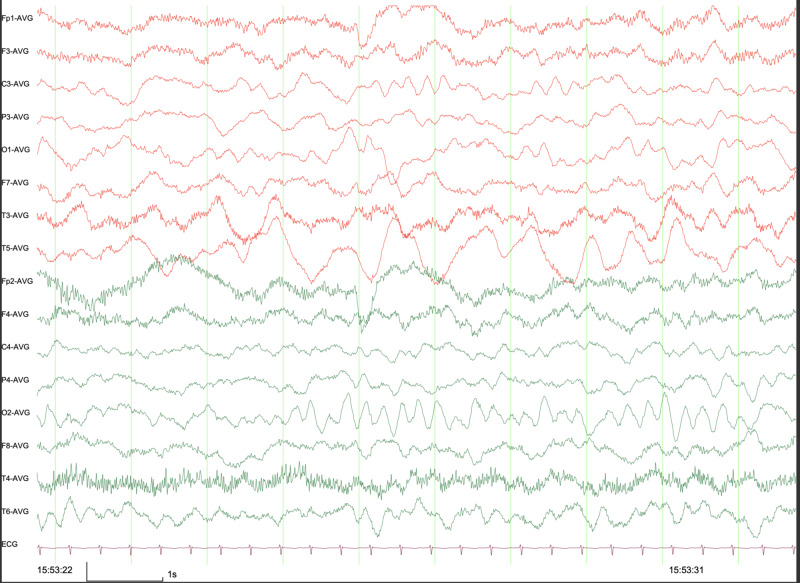
EEG of a 6-month, 3-week old female demonstrating asymmetrical background with left sided higher amplitude delta activity and slowing at the left-parieto-occipital areas.

Abdominal ultrasound was normal. ECG showed left ventricular hypertrophy (Figure 2). A transthoracic echocardiogram showed no abnormal findings, a transesophageal echocardiogram was recommended for better visualization but was felt not to be feasible due to her age. EEG showed asymmetrical background with left sided higher amplitude delta activity and slowing that was appreciated at the left-parieto-occipital areas (Figure 3). EEG reflected a potentially epileptogenic disturbance of neuronal function and indicated an increased risk for focal seizures.

The patient was discharged on levetiracetam 80mg every 12 hours po. She was also prescribed lorazepam 1 mg oral as a rescue medication. A follow up appointment was booked and a request for an outpatient MRI was made. It was also recommended that the mother be screened with a coagulation profile.

**Figure 4:**
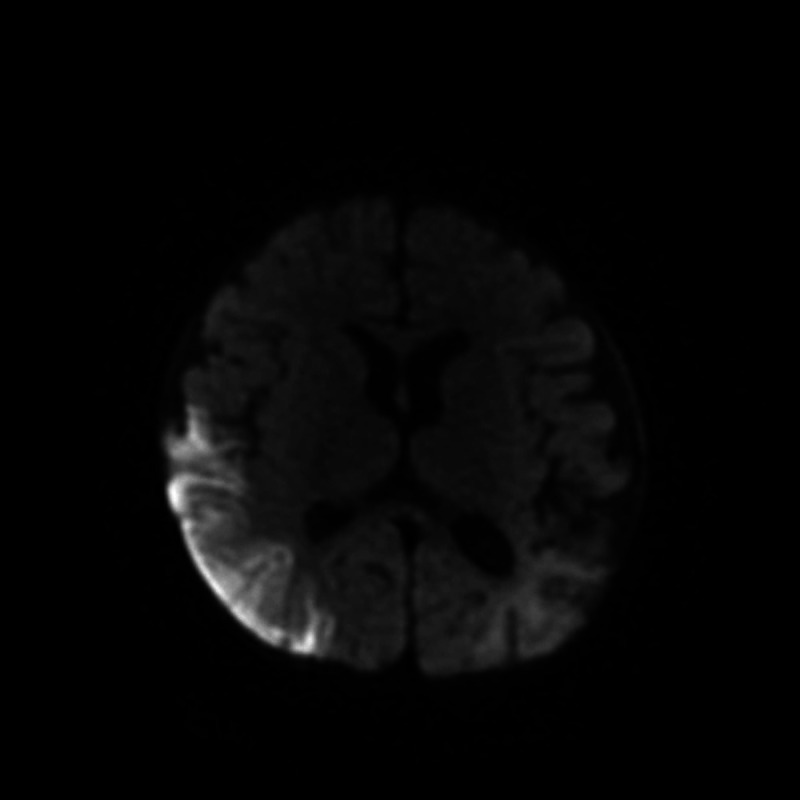
MRI of a 7-month, 3-week old female demonstrating a right-sided parietal lobe infarct.

One month later, the patient presented to her local hospital with status epilepticus. She was given a dose of phenobarbital (20 mg/kg/dose) then Keppra 10 mg/kg/dose at which point she stopped seizing. CT and MRI showed a new right-sided parietal lobe infarct (Figure 4). Her blood work once again did not indicate any cause for the new stroke. Five days later, she was transferred to the closest children’s hospital. She did not have any more seizures during her admission. She was on 80 mg of Keppra orally, twice a day. She was eating fine and was very active. Her mother did note that she used her left side less and was not grasping things with her left hand. 

Upon examination on admission to the children’s hospital, she was responsive and alert. She had a head circumference of 44 cm putting her in the 70th percentile. Her weight put her in the 60th percentile. Her motor examination was normal except for on her left hand and arm on which she had a 3/5 strength on her elbow and shoulder flexion and was unable to grasp objects. There was no facial drop. Her hearing and vision were normal, red reflex was present and there was no nystagmus. Her cardiovascular, respiratory, abdominal and dermatological exams were all normal.

Upon admission to the children’s hospital cardiology, ophthalmology, hematology, neurology and physiotherapy were all consulted. Cardiology performed an echocardiogram which had all normal findings. Ophthalmology did a complete eye exam for which there was no significant findings. Hematology did hypercoagulable studies for which she came back as normal. Her MRI also didn’t show any vasculitis. Neurology ruled out hematological causes and suggested the metabolic genetic team rule out other metabolic causes for stroke. Said team was consulted for a query of mitochondrial disease that could cause bilateral strokes at different timing. A central CSF culture, amino acid plasma study, CSF cytology, carnitine, CSF lactate, CSF protein, and CSF glucose were all normal. An amino acid chemical analysis was normal, except for a slight increase in cystine, but this is a routine, non-specific finding in CSF. Urine creatinine, CBC and ammonia were normal. Attempts were made but were unsuccessful in measuring serum lactate due to clotting. Mitochondrial DNA results were still pending at time of discharge. Hence, she was put on co-enzyme Q10 5mg/kg/day in three doses, riboflavin 100 mg daily and alpha lipoic acid 50mg daily until her results became available. Physiotherapy decided they could sign of her after the first day as they believed she would regain strength with daily exercises.

At the time of discharge four days following admission, the patient had regained some strength in her left arm and was able to grasp objects. There were no issues during her admission to the children’s hospital. Follow up was coordinated with the patient’s local neurologist.

**Figure 5:**
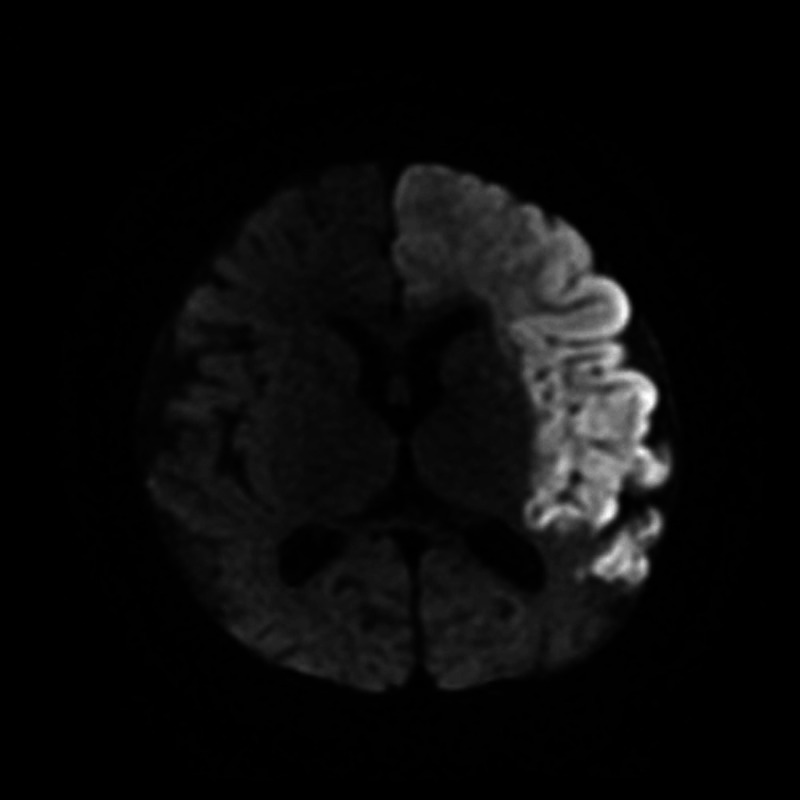
MRI of a 10-month, 3-week old female demonstrating extensive changes of the left hemisphere.

**Figure 6:**
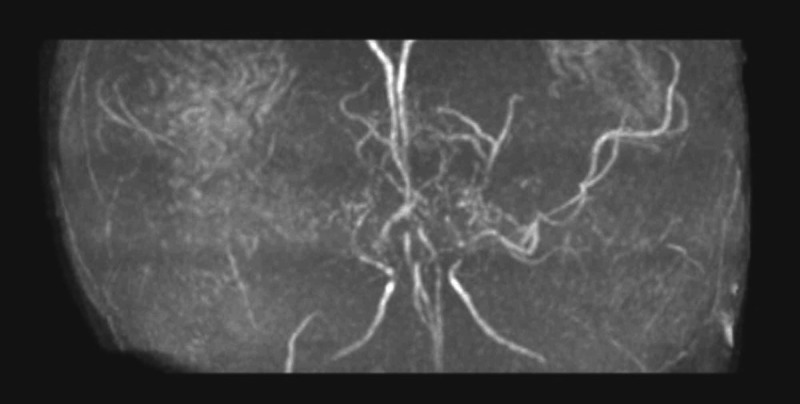
MRA of a 10-month, 3-week old female demonstrating severe stenosis of the terminal/supraclinoid carotid arteries bilaterally, a large number of small vessels in the supraclinoid region, extending into the sylvian fissures and posteriorly into the quadreminal cisterns primarily all along the circle of Willis.

Approximately three months later she had another stroke which resulted in extensive changes of the left hemisphere on MRI (Figure 5). Magnetic resonance angiography (MRA) also was consistent with MMD as it showed severe stenosis of the terminal/supraclinoid carotid arteries bilaterally, as well as a large number of small vessels in the supraclinoid region, extending into the sylvian fissures and posteriorly into the quadreminal cisterns primarily all along the circle of Willis (Figure 6). Around this time, the diagnosis of early onset MMD was made due to the MRA results and a completely novel de novo mutation in the RNF213 gene. It was recommended that bilateral encephaloduroarteriosynangiosis (EDAS) be considered. It was also recommended that she be monitored for signs of renal stenosis.

Five months after her initial presentation, she underwent uncomplicated bilateral EDAS procedures. In the following year, there have been no reported episodes suggestive of strokes. In addition to the left hemisphere changes from the stroke that occurred one month before her surgery, there was also right hemisphere atrophy present, as well as a left side subdural hematoma with no mass effect. She has remained on Keppra 400mg twice a day and aspirin 20mg once daily, and has been attending physiotherapy. She is feeding with no issues and is growing well. She has developed no new symptoms. On exam, she is well looking. She has no facial asymmetry and extraocular movements are normal. Her cranial nerve exam is grossly normal. She continues to have skull softening to the right side of vertex. Her right hand is in a fist-like position, and she has minimal use of the digits on this hand. Additionally, her right foot is in an externally rotated position when she walks, and she tends to drag it. Physiotherapy has been addressing this and she is showing some improvement. She is otherwise able to ambulate appropriately for her age. She also has hyperreflexia and hypertonia of her right arm. The remainder of her exam shows normal power on her left side with deep tendon reflexes being +2.

## Discussion

It is rare but not unheard of for moyamoya disease to appear in children in their first year of life [9-16]. The greatest incidence of MMD occurs in East Asia [17]. This patient adds to the small list of children who present so young with this disease, and is particularly rare as she is Caucasian. Although MMD can present later in life, the prognosis is known to be worse for those who are diagnosed at a young age [18]. Studies in Asian populations have shown a high association with the RNF213 gene on chromosome 17q25.3, which corresponds with our patient's completely novel de novo mutation in the RNF213 gene [19]. 

The natural history of moyamoya disease is associated with morbidity and mortality as the disease manifests in transient ischemic attacks, often leading to cerebral infarcts [17]. Due to the fast progression of MMD in young patients, research has shown that early revascularization is indicated to minimize damage done by the progression of the disease in the young population [18]. In one study patients who underwent revascularization surgery within 3 months after the onset of MMD had a lower incidence of preoperative infarctions [18]. This same study also showed no difference in the number of surgery-related infarcts between age groups when comparing children under the age of 17, showing the relative safety of the procedure at young ages [18]. 

Although our patient was able to have EDAS performed bilaterally as she did well during surgery, for those who are unable to have both sides operated on during the same surgery the question is which side should be operated on first. It has been recommended that in cases of small infarct that the affected side be operated, but in cases where there is a large infarct, the contralateral side be operated on first to attempt and preserve one healthy hemisphere [18].

The goal of the revascularization surgery is to protect the brain from further infarcts and attempt to reverse the ischemia caused by MMD. EDAS is the most commonly used surgical technique to treat MMD in children [19]. This has proven to be a relatively safe surgery with a perioperative complication rate of 3.5-7.7% and a post-operative complication rate of 4.2% [20]. Even with surgery, poor outcomes are predicted for patients who present with MMD at a young age, and/or have postoperative ischemic events. However, in a recent study, 86% of children aged 6 months to 18 years old had an independent life with no significant disability following their EDAS procedure [20]. 

## Conclusions

Our female infant Caucasian patient was diagnosed with early onset MMD due to magnetic resonance angiography showing severe stenosis of the terminal/supraclinoid carotid arteries bilaterally with moyamoya vessels, and a completely novel de novo mutation in the RNF213 gene. This case report reinforces the importance of keeping Moyamoya disease on the differential for children of all ages and ethnicities presenting with unexplainable stroke and seizures. A quicker diagnosis utilizing MRA may help reduce the time between first presentation and treatment with EDAS, therefore increasing the patient’s chances at decreased morbidity and mortality.
